# “Compassionate City” in Patients with Advanced Illnesses and at the End of Life: A Pilot Study

**DOI:** 10.3390/ijerph20032234

**Published:** 2023-01-26

**Authors:** Silvia Librada-Flores, María Jesús Pérez-Solano Vázquez, Miguel Ángel Lucas-Díaz, Zacarías Rodríguez Álvarez-Ossorio, Emilio Herrera-Molina, María Nabal-Vicuña, María Dolores Guerra-Martín

**Affiliations:** 1 New Health Foundation, 41004 Sevilla, Spain; 2 Palliative Care Team, Arnau de Villanova Hospital, 25198 Lleida, Spain; 3 Department of Nursing, University of Sevilla, 41009 Sevilla, Spain

**Keywords:** community networks, palliative care, quality of life, public health needs assessment

## Abstract

Objectives: To evaluate, in a Compassionate City pilot experience (Sevilla), the impact results on health in a population of people with advanced illness and at the end of life. Methods: The project was undertaken in Sevilla, Spain, between January 2019 and June 2020. A longitudinal, descriptive study was conducted using a longitudinal cohort design with two cross-sectional measurements, pre and post intervention. All patients who entered the program on the start date were included. The networks of care around people with advanced illness and at the end of life, palliative care needs, quality of life, loneliness, anxiety, depression, caregivers’ burden and family satisfaction were evaluated. The interventions were conducted by community promoters assigned to the “Sevilla Contigo, Compassionate City” program. Results: A total of 83 people were included in the program. The average number of people involved in care at the beginning of the evaluations was 3.6, increasing to 6.1 at the end of the interventions. The average number of needs detected at the beginning was 15.58, and at the end of interventions, it was 16.56 out of 25. The unmet needs were those related to last wishes (40.7%), emotional relief (18.5%), entertainment (16%), help to walk up and down stairs (8.6%) and help to walk (6.2%). A total of 54.2% showed improved loneliness in the final evaluation. Out of 26 people evaluated for pre and post quality of life, 7 (26.9%) improved their quality of life in the general evaluation and 5 (19.2%) displayed improved anxiety/depression. A total of 6 people (28.6%) improved their quality-of-life thermometer scores. A total of 57.7% of caregivers improved their burden with a mean score of 17.8.

## 1. Introduction

Compassionate Communities at the end of life have been conceived as a model of social innovation that complements palliative care towards integrated health, social and community care [1,2,3,4]. In addition, compassion has been shown to improve the quadruple aims of improving patient experiences, population health, professional experiences and organizational effectiveness [5].

In recent decades, there has been an emerging development of Compassionate Communities and Cities designed with the aim of involving society in the care and support of people who are in a process of advanced illness and/or at the end of life, being diagnosed with cancer or other pathologies [6,7]. These programs are oriented to the characteristics of the Charter for Compassion by Public Health and Palliative Care International (PHPCI) [8]. Several methods have been designed that have allowed their implementation in different parts of the world [3,9,10]. Even so, there is some variability in the implementation and evaluation models of Compassionate Communities and Cities [10].

Most of the initiatives launched have been aimed at raising awareness and educating society about the importance of caring for and accompanying these people [6,7,11] and, to a lesser extent, mobilizing community resources and intervention processes [10].

As Abel et al. [12] and Librada et al. [13] have already described, the development processes of Compassionate Communities and Cities must be oriented towards the processes of awareness and the formation of society, the involvement of key agents, coordination of resources, and the development of structures, processes and protocols for community intervention.

Community intervention experiences are unusual and differ from one another in terms of impact results. Studies have been conducted measuring the economic impact of the development of public health and palliative care initiatives, such as the experience of the Frome, a Compassionate City [14], which have shown a reduction in hospital and emergency readmissions thanks to the involvement of the community. Luzinski et al. [15] analyzed the efficiency cost of community intervention processes, producing an average saving of USD 93,000 per year for the group of beneficiaries of the community case coordinator program.

Other initiatives have been shown to improve quality of life in people at the end of life with volunteering experiences, improve loneliness and increase community involvement [16], and reduce caregiver burden [17].

In the processes of community intervention, the first goal is generating care networks that allow the meeting of the needs of people with advanced illness and at the end of life thanks to the involvement of the community [18,19]. For this, the RedCuida protocol [20] was designed, which allows the detection of the needs in the person and generation of community care networks, while evaluating the improvement in the quality of life and well-being of these people and their families. The RedCuida protocol is conducted by the community promoter and is mainly based on the activation of community, internal, external and community networks of care to help in the distribution and assignment of responsibilities that allow the care and support needs of these people and their families to be covered. [21].

The “Sevilla Contigo, Compassionate City” program of the New Health Foundation is the first pioneering program in the world to implement the “Todos Contigo” method [9] and to use the RedCuida protocol [20] as a process for detecting needs and generating networks of care.

The objective of this study was to evaluate, based on this pilot experience, the impact results on health in a population of people with advanced illness and at the end of life, diagnosed with cancer and other non-oncological diseases, in terms of quality of life and well-being, anxiety/depression and loneliness. As well as evaluating the care and support networks around these people, the emotional/physical caregiver burden and the satisfaction of caregivers and family members were evaluated.

## 2. Materials and Methods

### 2.1. Studio Design

A longitudinal, descriptive study on a population of people with advanced oncological or non-oncological illness and/or at the end of life referred to a community intervention program “Sevilla Contigo, Compassionate City” between 1 January 2019, and 30 June 2020.

### 2.2. Study Population

Inclusion criteria: People living in several districts of the city of Sevilla (684,234 inhabitants) with oncological or non-oncological illness, advanced and/or at the end of life who had palliative care needs that could be covered by the community, by a palliative care team and by a community promoter.

Exclusion criteria: People in a very advanced state of their disease with a life expectancy of less than one week. A patient was considered to be discharged from the program when they had died or voluntarily resigned from the program, or moved to an area where there was not a program intervention.

The value of data loss was estimated to be 20% from the inclusion criteria and 15% from the possible disclaimers, loss of information or non-response of the population during the intervention process.

### 2.3. Intervention Process and Variables

The intervention process of people referred and beneficiaries of “Sevilla Contigo, Compassionate City” program was followed in accordance with the RedCuida protocol [20] using the assessments and scales defined in the protocol. The community intervention was conducted by a community promoter who is the person in charge of identifying needs of the patients and generating care networks. The community promoter collaborated with professionals (primary care, palliative care team, social services, key agents) who referred these patients to the “Sevilla Contigo” program. After the referral, based on the previously defined inclusion criteria, the community promoter contacted the patient and her family and arranged a first face-to-face appointment at the usual place of care (home, hospital, or residential center). In an initial assessment, the community promoter, through a series of assessment scales, identified care needs for the development of daily life and needs for care and accompaniment. She also identified the number of people involved in care throughout the disease process. Based on this information, she assigned, in successive assessments, the different people identified in the network to the needs of the patient, in order to be able to cover those needs.

The variables used for the study were the following:
Sociodemographic characterization of people referred and beneficiaries of the program.Sex (Man/Woman).Average Age.Diagnosis (Oncological/Non-Oncological).Referral to the program.Number of people referred to the program.Origin of referral (Primary Care, palliative care teams, social services, key agents, community network, direct request by family member).People in a situation of last days to the date of referral to the program.

Inclusion in the program, scope of care and state of the population.
Number of people included in the program.Care setting (home, hospital, residential center).State of the population:○Active: patient who meets criteria and has active follow-up by the promoter throughout the entire intervention process.○Passive: patient who, being within the program, sees their needs satisfied thanks to the intervention of the promoter and the network and who passes to this passive state where less follow-up is conducted (biweekly by phone) to verify that the needs have not changed and continue to be covered, and to whom at least one face-to-face visit is made per month.○Resignation: person who decides not to continue in the program.○Transfer: person who leaves the program due to transfer to another area where the program is not offered.○Deceased: person who dies while in the program.Place of death.Median survival of patients included in the program.


Social assessment of beneficiaries of the program.
People included in the program with social support networks (internal, external and/or community).
Sociodemographic characterization of main caregiver.
Number of people with main caregiver.Sex (Man/Woman).Average age of main caregiver.Relationship: Father/Mother, Spouse, Son/daughter, Brother/sister, Cousin, Nephew/niece, Grandson/daughter, Son-in-law/Daughter-in-law, Friend, Professional Caregiver, Domestic Employee, Other.
Characterization of initial network and final support network (internal, external and community).
Number and average number of people in the initial network.Number and average of people involved in the initial network.Number and average number of people in the final network.Number and average number of people involved in the final network.Average increase in the networks involved from start to finish.


Quality of Life and Well-being Impact.Number and average needs of patients in the initial and final assessment.Number and average of satisfied needs of patients.
For the application of the RedCuida protocol, the Barthel scale [22] and Lawton and Brody [23] were used and 7 types of emotional and support needs were added [20] to learn about the need for help in each activity and if this need was being met or not met by some member of the community. For each type of activity, the following was collected:
You do not need (you do not require) help from the community to conduct the activity.Satisfied:
○YES: When a member of the community helps you in the development of the activity and the profile of the person who meets the need is identified. To score this field, the need must be 100% satisfied by the community member(s). To evaluate this field, the promoter makes the assessment, whenever possible, with the main caregiver, taking into account, in case of overload, what kind of help is needed, and recording said needs as “unsatisfied” when it is necessary to incorporate people from the network for your complete satisfaction.○NO: When you need help, you do not have any member of the community, or those you have do not reach 100% of the help required in the development of such an activity, either because your needs are greater or because the person covering them is overloaded and needs help. In this case, unsatisfied must always be marked and it will be part of the intervention process to identify another member of the network who satisfies it.


Assessment of Loneliness (ESTE II Scale) [24].
Percentage of patients who improve loneliness. The total score of the scale ranges between 0 and 30 and is obtained by adding the score of each of the items on the scale. There are 3 levels of Social Loneliness depending on the score obtained: Low → 0 to 10 points; Medium → 11 to 20 points; High → 21 to 30 points.
Quality-of-Life Assessment (EUROQOL-5D) [25].Percentage of patients with improved quality of life.Percentage of patients with improved anxiety/depression.In each dimension of the EUROQOL-5D scale, the severity levels are coded 1 if the response option is “(I have) no problems”; with a 2 if the answer option is «some or moderate problems»; and with a 3 if the answer option is «many problems». The second part of the EUROQOL-5D provides a complementary score to the descriptive system of the self-assessment of the individual’s state of health, using a vertical VAS scale of 20 cm and millimeters, ranging from 0 (worst state of health imaginable) to 100 (best imaginable state of health). The combination of the values of all the dimensions generates a 5-digit number, with 243 possible combinations of health states, which can be used as profiles. The average quality of life is obtained, among all these combinations.Main Caregiver Burden (Reduced Zarit Scale) [26].Number of main caregivers with burden/not burdened in the initial and final evaluation.Number of main caregivers who improve their burden in the final assessment.The maximum possible score is 35 points. An equal result, or less than 17 points, would indicate that there is no burden, and a value bigger than 17 is the opposite.Family satisfaction.Satisfaction with the community intervention process.The satisfaction scale is conducted by a person other than the one who conducted the intervention process. It is conducted via a telephone survey of 8 items to the caregivers of deceased patients in which the caregiver responds to the survey by answering each of the questions with the scores: No, never (1), A few times (2), Sometimes (3), Quite a few times (4), Yes, always (5).

### 2.4. Information Sources, Data Collection and Processing

The variables of the study were compiled in a data collection notebook by the community promoter in each of the interviews and scales were applied to the beneficiaries of the program and their families. A database was designed in Microsoft Access for the recording of the variables under study and their subsequent data processing.

### 2.5. Statistical Analysis

A descriptive analysis of all the variables related to the needs of the patients and the profiles of the people involved in the network was conducted. The data before and after the detection of needs, generation of care networks and start and end assessments were analyzed and described. This analysis included measures of central tendency (mean, median, standard deviation), 95% confidence intervals for the mean, contingency tables, and frequency analysis.

### 2.6. Ethical Considerations and Data Protection

The study was approved by the Ethics and Research Committee of the Virgen del Rocío University Hospital and the Virgen Macarena Hospital of the Andalusian Health Service of the Junta de Andalucía, Spain (CI 1020-N-17), in June 2018. The study used an informed consent approved by the mentioned Committee.

All beneficiaries of the program, and their main caregivers, were informed verbally and in writing of the intervention process, signing an informed consent for participation in the program and for the provision of their data for analytical and research purposes. To guarantee the confidentiality of the data, they were codified and encrypted so that they could not be identified. The questionnaires were coded with an alphanumerical identifier in a separate database independent of the one containing the patient identification data.

## 3. Results

### 3.1. Referral to the Program

From 1 January 2019 to 30 June 2020, 104 people were referred to the program (53.8% men, 46.2% women). A total of 49 had an oncological disease (47.1%), 47 non-oncological (45.2%) and 8 without a specification of the diagnosis (7.7%). The average age of the people was 74.3 years (svtp 14.2).

Of this population, 16 people (15.4%) refused to enter the program because their needs were covered by their family network or because they needed another type of help other than that of the program. A total of 5 people (4.8%) were in a last-day situation when they were referred to the program.

The origin of the referral to the program is represented in Table 1.

### 3.2. Population Included in the Program

Finally, 83 people (79.8%) were included in the program. There were 45 men (54.2%) and 38 women (45.8%), with an average age of 74.5 years (svtp 14.4). There were 41 cancer patients (49.4%) and 40 non-cancer patients (48.2%). No diagnostic data was obtained for 2 of the people included in the study (2.4%). A total of 78.3% of the patients included in the program belonged to the San Pablo-Santa Justa district, 20.5% to the Macarena district, and 1.2% to the Nervión district.

### 3.3. Scope and Status of the Population at the End of the Study

The care environment of the people included in the program was 80.7% home care, 13.3% hospital care and 6% in residential centers. The average number of days spent in the program was 133 days (home setting: 151 days; hospital setting: 57 days; residential center: 64 days). The average length of stay for cancer patients was 170 versus 104 days for non-cancer patients.

During the study period, 36 people (43.4%) died, 12 abandoned the program (14.4%) and 3 were transferred to other services or locations (3.6%). From the remaining 32 people, 17 remained active in the program (20.5%) and 15 passives (18.1%).

**Figure 1:**
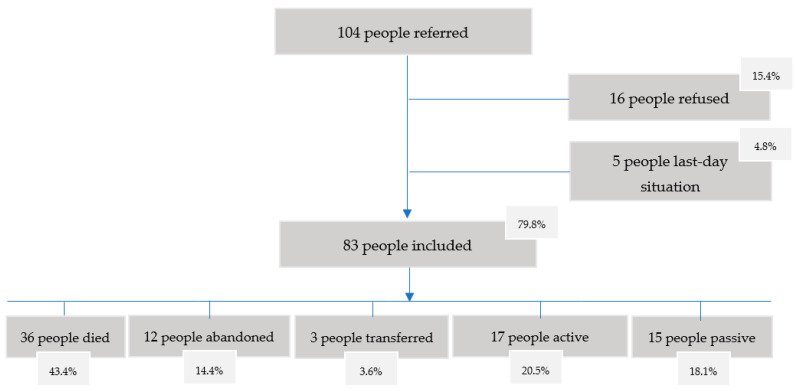
Inclusion process flowchart.

From those who died in the program (*n* = 36), 11 died at home (30.6%), 24 in hospital (66.7%) and 1 in a residential center (2.8%). The average survival of patients who died in the program was 21.5 days. The inclusion process is represented in the following Figure 1.

### 3.4. Social Assessment of Beneficiaries of the Program

At the time of inclusion in the program, 94% (*n* = 78) of the patients had a social support network. A total of 6% (*n* = 5) did not have a social support network.

### 3.5. Sociodemographic Characterization of the Primary Caregiver

A total of 85.5% of the patients had a primary caregiver (*n* = 71). Of these, 94.4% were direct relatives and 5.65% non-relatives. The sociodemographic profile of the main caregivers was 66.2% women (*n* = 47) and 33.8% men (*n* = 24) with a mean age of 57 years (svtp 15.24). The kinship of the caregivers corresponded to 42.3% being a son/daughter, 36.6% spouse, 5.6% brother/sister, 4.2% niece/nephew, 2.8% professional caregiver, 2.8% son-in-law/daughter-in-law, 1.4% mother, 1.4 cousin, 1.4% friend and 1.4% domestic employer.

### 3.6. Initial Network and Final Support Network

At the time of inclusion in the program, an assessment of the initial network was made in 78 patients. The total number of people included in the initial network was 420 people, including 72.9% family members and 27.1% non-family members (with 17.6% from the external network of friends, neighbors, etc., and 9.5% from the community). The average number of people in the initial network was 5.4 people. Of these 420 people, 285 (67.9%) were directly involved in care and support, resulting in an average of 3.6 people involved in the initial network.

The total number of people included in the final network was 487, with 60.6% being family members and 39.4% non-family members (including 24.0% from the external network and 15.4% from the community). The average number of people involved in the final network was 6.1, assuming an increase in the networks involved from the beginning to the end of 2.5 people (Table 2). An analysis of the increase in care networks was conducted for 57 people who were the same people assessed at the beginning and at the end. Of this population, in 32 people (56.1%), their care network increased; in 22 people (38.6%), the network was maintained from start to finish; and in 3 people (5.3%), the network worsened. A total of 32 volunteers who accompanied 25 patients were involved in their care (Four of them had two volunteers assigned).

### 3.7. Impact on Quality of Life and Well-Being

The initial and final needs of the beneficiaries (Barthel, Lawton, Brody adapted).

The community promoters evaluated a total of 25 needs (10 basic activities of daily life, 8 instrumental activities of Daily Life and 7 other types of needs).

Upon entry into the program, a needs assessment was made for a total of 81 people (97.6% of the total population). The average number of needs per person, among the 25 needs assessed, was 15.58 (representing 62.3% of the needs assessed). These detected needs were covered by members of the network in 91.5% of the cases. On the other hand, 8.5% of the needs identified in these people were not covered by members of the network in this initial assessment.

The needs not covered in the initial assessment were those related to the preparation of last wishes (40.7%), followed by emotional release (18.5%), entertainment (16%), help to go up and down stairs (8.6%) and help in ambulation (6.2%) (Table 3).

The assessment of needs in the final network was made for a total of 62 people (76.5% of people with a final assessment with respect to the people assessed in the initial network). The average of needs per person was 16.56. A total of 6.9% of the needs of these people were left uncovered in the final network (Table 4).

### 3.8. Assessment of Loneliness (ESTE II Scale)

Loneliness was assessed in 57 patients at the initial assessment.

At the beginning of the intervention, 68.4% of the people had some type of loneliness (64.9% with a medium level of loneliness on the scale and 3.5% with a high level) (Table 5).

Evaluations of loneliness at the beginning and end of the interventions were completed in a total of 24 people. For these 24 people, the mean loneliness score in the initial evaluation was 12.75, and in the final evaluation, there was an average of 12.12. (Mean difference −0.62500). A total of 54.2% (13 people of the 24 evaluated at the beginning and end) reported that their perception of loneliness improved in the final evaluation. A total of 100% stated that they felt more accompanied after the work of the community promoter.

### 3.9. Quality of Life (EUROQOL 5D)

The quality of life was evaluated in 73 people in the initial evaluations and in 26 in the final evaluations. The average quality of life in the patients who were evaluated at the beginning of the intervention was 0.34 (0 worst quality of life–1 best quality of life on the scale). The average quality of life in the final assessments was 0.36 (Table 6).

Out of the 26 people evaluated in the initial and final assessments, 7 (26.9%) improved their scores on the general scale and 5 (19.2%) improved their anxiety/depression scores. A total of 6 people (28.6%) improved their quality-of-life thermometer scores.

### 3.10. Burden of Primary Caregivers (ZARIT Reduced Scale)

The evolution of caregiver burden using the reduced Zarit scale was measured, in the initial and final assessments, for a total of 26 main caregivers. Of these, at the beginning, 64.4% (*n* = 17) of the caregivers presented overload with a mean score of 20.8 on the reduced Zarit scale. In the final evaluation, 34.6% of the caregivers (*n* = 9) presented burden, and 57.7% improved their burden with a mean score of 17.8.

### 3.11. Satisfaction of the Community Intervention Process

**Figure 2:**
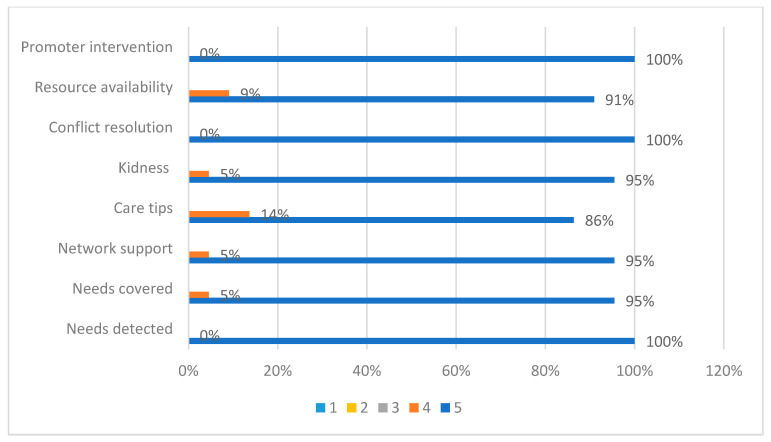
Satisfaction survey of relatives of patients cared for in the “Sevilla Contigo” program.

Satisfaction was assessed in a total of 22 caregivers of deceased patients in the program. A total of 100% of the people who answered the survey reported a very high level of satisfaction with an average satisfaction survey score of 39.5 out of 40 (Figure 2).

## 4. Discussion

After a period of development of the “Sevilla Contigo, Compassionate City” pilot experience, it has been possible to evaluate the impact on the health of the beneficiary population of the program thanks to the process of detecting needs and generating care networks through the RedCuida protocol [20]. This process has been possible thanks to the intervention processes developed by community promoters, professional figures assigned to the program, and is based on the activation of the community, internal, external and community networks to help in the distribution and assignment of tasks that cover the care and support needs of these people and their families.

The period of analysis for the evaluation of the impact on the population was from 1 January 2019 to 30 June 2020, in which time, due to the situation of the COVID-19 pandemic [27], in-person visits to the homes of patients were suspended and follow-up was continued by telephone. However, these interventions are still being evaluated, especially the way in which the community, organizations and volunteer entities have been responding to this situation. Qualitatively, some of these actions have been highlighted, such as support for families in mourning when it was not possible to say goodbye, telephone support for people in a situation of loneliness due to social isolation, help with some of the instrumental activities of daily life such as shopping, and advice and recommendations to professionals from other neighboring countries who, in the same way, had to reinvent support and accompaniment processes from a social distance [28]. The evaluation of the processes of community intervention in this time of pandemic, as well as the impact evaluations, have not been completed yet because, to this day, interventions continue to be conditioned by this situation.

For a population of 135,310 inhabitants, which is the one that would correspond to the districts where the program operates, around 1420 people would die annually, according to the mortality rate of the districts (9.74, 11.12) [29]. According to estimations by McNamara et al. [30], 70% of this deceased population in these districts would be likely to receive palliative care, and out of these, around 40% would need specialized palliative care. Therefore, we would be talking about a population of around 400 people who could have been referred to the “Sevilla Contigo, Compassionate City” program. According to these estimations, the program has been offered to 26% of the population that could be considered a beneficiary of the program, and although this number has been increasing, referrals are still rare. One of the greatest contributions of the program has been the participation of agents from the health and social sectors in integrated commissions that have facilitated referral, although this type of structure still needs to be strengthened to guarantee a coordinated and integrated response to the needs of these people and their families [31].

Being a longitudinal study, and due to the characteristics of the population, the probabilities of non-inclusion were estimated at 20% and those of abandonment during the study period were estimated at 15%. Therefore, the percentage of rejections or non-inclusion is considered appropriate to the forecasts that were made according to the type of study [32].

The population referred to a palliative care program is usually oncological, even though the prevalence of diseases is 70% non-oncological and 30% oncological [30]. In this case, the proportion between oncological and non-oncological patients was very similar, in part because the identification of the people requiring a palliative care program is increasingly being improved, since the inclusion criteria were extended to people with advanced illness, high dependency and disability, which are profiles usually attended to by social workers from the city council’s social services who actively participated in the referral.

One of the objectives of palliative care is to provide care in the place of preference of patients and their families [33]. The home is considered the preferred place of care for most people with advanced disease and at the end of life, but it is also the place where the greatest social needs are found. Therefore, if there is a palliative care team that provides home care and support and as long as there is a good network of care, this place is considered the ideal place for the provision of community-based palliative care. From the “Sevilla Contigo” program, 80.7% of people were cared for in a home environment, 6% in residential centers (considered the home of these people) and 13.3% in a hospital environment. These data coincide with those of other studies where the preferences for the care of patients at the end of life in the home setting are between 70 and 90% [34]. It can be interpreted that the place of home care is an indicator of the satisfaction of patients and relatives, but it is not related to the preferred place of death, since these preferences can change throughout the process.

Increasingly, the chances of dying at home decrease with age; the older the person, the greater the chance of dying in hospital. This relationship is in turn related to the scarcity of networks, with not wanting to be considered a burden to others [35], with feeling safer in hospital or due to the lack of provision of services at home [36]. In the case of people who died within the program something similar happened. More deaths occurred in hospital (66.7%) than at home (30.6%) or residential center (2.8%), despite the fact that care at home was the most preferred. Thus, it would be necessary to consider as an indicator of quality in the person and their family, the ability to comply with the preferences of these people regarding the place where care is provided and the place where they die.

The median survival of patients treated in the “Sevilla Contigo” program was 21.5 days, slightly higher than the median number of days of people who died in palliative care programs, which was 19 days [37].

In the absence of other studies that have measured the relationship between community intervention processes and survival in a palliative care program, it has not been possible to show whether this effect could be improving thanks to the creation of intervention structures such as integrated commissions or to the actions of social awareness and training that cause greater knowledge about the referral pathways to a palliative care program.

Regarding the social assessment and characterization of the networks of the people included in the study, the majority (94%) had a social support network at the beginning of the program, with a greater proportion of people who had only an internal network formed by close relatives (67.9%), since in most cases these people had a primary caregiver (85.5% of cases). These data coincide with the study by Díaz, Redondo and Librada, 2016 [38], where the figure of the caregiver was represented by 90% of the cases, and other profiles could be observed, as has also been possible to demonstrate in this study. In addition to the internal network, 16.7% had other external networks such as neighbors, friends, or acquaintances, and 6.4% had broader networks that included, in addition to internal and external networks, the community. A total of 6% of the people included in the program did not have any social network (*n* = 5). These initial assessments conducted by the community promoter were key to identifying the existing people involved in the care, programming the intervention plans for the generation of community networks and being able to compare the networks at the end of the intervention process.

The profile of the main caregivers of the people in the “Sevilla Contigo” program also coincided with those of other studies. This profile is related to family structures, which continue to be the main sources of care in most developed countries [39,40,41].

The program in Sevilla made it possible to double the care network. This increase highlights the importance of these intervention processes with the community and the way in which the figure of the community promoter allows the involvement of new profiles. These results have made it possible to corroborate the hypotheses of Abel and Horsfall [18,19], about the increase in care networks thanks to the existence of figures that act in these processes, and the possibility of being able to involve other members of the community, such as those detected in the studies initiated by Díaz, Redondo and Librada [38], where the tasks that can be performed by each of these profiles in the processes of advanced disease and end of life were identified.

The needs, as classified by Ferris and Librach [42], are multiple and different in each person and cannot be covered solely by the network of professionals who attend the person in this process, even more so when the needs are, for most of a person’s time at the end of life, of a social nature. The people in the program presented an average of 15.58 needs (of the 25 evaluated) at the beginning of the interventions, being supported by the members of the initial network in 91.5% of the cases. Most of the needs not covered in the initial network corresponded to last wishes (40.7%), followed by emotional relief (18.5%), entertainment (16%), help to go up and down stairs (8.6%) and help in wandering (6.2%). This typology of detected needs coincides, for the most part, with the preferences for care of people at the end of life collected by Borgstrom in 2020 [43], where the aspects to be taken into account at the end of life are contemplated: being treated with dignity and respect, being free of pain and other symptoms, being in a familiar environment and in the company of family and/or friends, being prepared for the moment of death, expressing feelings, giving meaning to death and life, and feeling safe from care until the last moment. Therefore, a beneficial contribution of the RedCuida protocol was to be able to identify these assessments of the emotional and support needs in these people and to be able to be attentive to these preferences in the population [20,43].

It should be noted that, for this study, the assessment of needs was limited to those related to the basic and instrumental activities of daily life and to a series of emotional and support needs that were adapted in the RedCuida protocol. This selection is due to the fact that the intervention processes with the community aimed at addressing this type of needs thanks to the community and complement this action with those of professionals who address other types of physical, psychological, emotional or spiritual needs in a multidisciplinary manner. [44].

Regarding loneliness, even though its measurement is complex due to the objective and subjective components that define it, we wanted to measure and address the way in which loneliness is perceived as lower thanks to the community. Loneliness causes inactivity, depression, anxiety, and older people who are less lonely make greater use of health services such as consultation or emergency care. This frequency can be up to 50% higher than in the rest of accompanied people [45]. The perception of loneliness by the person themself and by professionals can help promote the mechanisms that allow the community to get involved. Thus, at the beginning of the interventions, 68.4% of the people showed some type of loneliness and at the end of the community interventions, and thanks to the involvement of the community, loneliness improved, with 54.2% of the people evaluated reporting having improved loneliness. The very presence of the community promoter figure throughout the intervention process also improved the perception of loneliness since the face-to-face visits and phone calls made them feel accompanied.

The quality of life and well-being in people with advanced disease and at the end of life is a situation that is continuously pursued and that is difficult to measure when trying to gather those factors that may be causing a state of discomfort in a person. The selection of a tool to measure quality of life can also be conditioned by the interventions that are going to be conducted on the patient. Thus, being able to put together a scale that brings together all the dimensions of well-being is complicated as well as inappropriate when the patient is in a highly complex situation [46].

The quality-of-life scales in palliative care have been designed to be simple, brief and help quickly identify those situations that are causing this problem. The choice of the EUROQOL 5D scale was determined by the fact that it is a tool that meets these characteristics of simplicity in the application of the scale and measures situations that could be improved thanks to the involvement of the community, such as support in daily activities or anxiety/depression improvement thanks to accompanying actions [47].

As occurs with the needs of patients at the end of life, which vary and increase due to the evolution and complexity of symptoms and the approach of death itself, quality of life, in this profile of patients, also tends to become worse [46]. Even so, it has been observed that thanks to the intervention processes with the community, an improvement in the quality of life was achieved in 26.9% of the patients.

Mobility, self-care, activity, pain/discomfort, and anxiety/depression states were improved for nearly all endpoints. It is true that the networks and accompanying actions cannot reduce a person’s pain from the physical point of view, but it has been proven that the perception of pain decreases as well as the values of anxiety/depression, which improved in all the final evaluations. A total of 54.8% of the patients initially presented anxiety and/or depression, and of these, 11.5% manifested it in a very severe way. At the end of the intervention, 42.3% presented anxiety and/or depression, seeing this improvement in 19.2% of the cases. According to Yanguas et al. [48], loneliness is a predictor of anxiety/depression. Thus, the decrease in anxiety/depression may in turn be conditioned by the decrease in loneliness in those patients who reported feeling alone and in whom this situation changed. Empathy, communication and listening also improve anxiety and depression and influence the well-being of people and their families [5], so they could also be conditioning this improvement as well as a compassionate approach and the desire of the community to alleviate the suffering of these people. Therefore, we can conclude that the support and accompaniment interventions by the community have had a positive influence on the improvement in the quality of life and well-being of these people. It would have been necessary to conduct correlation tests between these variables in order to statistically verify this relationship, but the variability of the sample has not allowed it to be compared. Finally, in the patients who were evaluated for their quality of life, they were also asked how they perceived their quality, and it was observed that 28.6% of the patients evaluated improved their perception of it, with the importance that this supposes. Not only had they improved their quality of life, but they were aware of it.

A total of 57.7% of caregivers improved their burden on the Zarit scale, going from a value of 20.8 to 17.8. This indicator is also related to the progressive increase in needs since, sometimes, although the network increases, as the number of needs to be satisfied and their intensity increase in parallel, it is more complex to lower the overload levels of the main caregivers, and a positive result is a slight improvement or even having maintained these levels at the end of the intervention.

Satisfaction is a quality indicator widely used at the end of life that expresses in a very close and sincere way the way in which a certain service has been provided, even more so when in the most difficult moments of a person and their family’s life, and we may feel this sense of being satisfied and grateful with the process [49]. The assessment obtained from these satisfaction surveys of the “Sevilla Contigo” program scored a satisfaction level of 39.5 out of 40 (very satisfied). These scores were given by 100% of the people who answered the survey. This result has motivated us to continue with the program and extend it to other towns and cities.

This study has been the first to analyze the health impact of a Compassionate Cities program in advanced illness and at the end of life in terms of improved quality of life and well-being, needs being met, decreased loneliness, decreased anxiety and depression, increased care networks, decreased caregiver burden and family satisfaction. To this date, there are no other similar studies to compare these results within populations with advanced disease and at the end of life.

It has been found that some of the intervention processes such as the circle of care tool have also shown good results in people with dementia or disability in improving quality of life and well-being, reduction in the overload of caregivers and reduction in the consumption of health resources for people with high support needs [50]. However, because the “Todos Contigo” method is currently being replicated in 16 cities in Spain, Portugal and Latin America [4,5,6], it is expected that these evaluations will be available in the coming years in the population at the end of life and will be able to achieve the results of this research. Therefore, far from pretending to be considered an isolated study of an isolated experience, we will try to show that thanks to the development of this model of community and integrated intervention, it will be possible to measure the impact of the implementation of the “Todos Contigo” method in those cities in which it is developing.

## 5. Limitations

Full evaluations of the development of Compassionate Cities have not been found to this date. For this reason, the possibility of evaluating a pilot project such as “Sevilla Contigo” was necessary to be able to measure the behavior of the structure, process and result indicators that allow for the measuring the impact on health and the improvement in the quality of life and well-being. On the other hand, there was a limitation in the selection of coverage areas, and having more professional figures such as community promoters, would have made it possible to increase the scope of the program in the city, reaching a greater part of the population, and consequently, obtaining a larger study sample.

One of the most important limitations of the study has been the inability to identify and manage referrals of the population to the program. The study population has been limited by referrals from professionals, and although coordination and referral structures have been developed that could be integrated into existing services in the city, the desired sample size has not been achieved. For this reason, more research would be needed to identify the beneficiaries of this program that would allow, on the one hand, covering a greater part of the population of Sevilla and, on the other, comparing the populations for each of the districts in which the investigation will be repeated.

The RedCuida protocol was designed to detect needs and to generate networks, but it does not describe a specific process of community intervention, so there could be a bias in the interventions of each of the promoters who conduct these processes with the family. The activity of the community intervention processes has not been analyzed since it has not been possible to compare them as there is no similar community intervention process in other populations. Even so, the activity of the community promoter was recorded for the internal evaluation of the resource and of the activity within the “Sevilla Contigo” program.

The analysis conducted in the initial and final evaluations were of a quantitative nature, although they have a qualitative interpretation that need to be studied in greater depth. The needs of people cannot be interpreted at the same level, nor considering that a need can be covered by a single person. The assessment of the needs that were covered by the network were considered by the promoter from the perception of the people who were being interviewed and their families.

The values of quality of life, satisfaction, decrease in the overload of caregivers and family satisfaction also accompany these results, but it cannot be interpreted that they improve only due to the intervention of the community promoter, since the care provided by the palliative care professionals, from health centers, social services or other key agents, also condition these results.

The investigations conducted with people who are at the end of life also suppose a limitation due to the very fact of the situation, since on some occasions, assessments cannot be conducted due to the conditions of the patients. Additionally, and more importantly, the initial assessments that start with a larger number of people end up with much smaller samples since they can only be compared with people who have died at the end of the study. For this reason, the populations were been comparable, and the study was limited to a descriptive analysis.

The results have allowed us to corroborate the starting hypothesis where community intervention processes improve the quality of life and well-being of people at the end of life, while allowing an increase in the care network and a decrease in the burden of caregivers, but these data, as a whole, were not been able to be compared with each other through variable correlation tests, because the sample, due to its own condition of being in an end-of-life situation, sometimes led to the evaluations not being completed, either due to the complexity of the patients when applying the scales, or due to the deaths of the population, which occur in different periods, causing the start and end evaluations not to be completed due to same reason.

Finally, one of the limitations of the study was not being able to obtain a control group that would allow the comparison of health consumption, and its associated costs, of community intervention processes vs. traditional care. This comparative study supposes the next line of investigation of the impact of Compassionate Communities and Cities. With these results, it is expected to be able to complete the evaluation of the impact on health and cost efficiency of the development of Compassionate Communities and Cities from the models of integrated care and from community-based palliative Care.

## 6. Conclusions

The “Todos Contigo” method and the systematic evaluation of the interventions for the development of Compassionate Communities and Cities allow for the verification of the improvement in the quality of life and well-being of the patients, a greater satisfaction of the family and their care networks, an improvement in loneliness, an increase in the care network and a greater number of needs satisfied by the network.

More research is required on community intervention processes and on mixed evaluation models that allow for a more detailed evaluation of the results of these interventions adapted to the preferences of these people and their families.

## Figures and Tables

**Table 1:** Origin of the referral to the program.

District	District 1. (San Pablo/Santa Justa)			District 2. Macarena		District 3. Nervión		Total
Origin	Cancer	Non Cancer	No Data	Cancer	Non Cancer	Cancer	Non Cancer	*n* (%)
Primary Care	27 (32.5%)	17 (20.5%)	7 (8.4%)	3 (15.0%)	9 (45.0%)			63 * (60.6%)
PC Team	6 (7.2%)	9 (10.8%)		4 (20.0%)	1 (5.0%)			21 (19.2%)
Social services	3 (3.6%)	11 (13.3%)	1 (1.2%)	1 (5.0%)				16 ** (15.4%)
Key agents	1 (1.2%)	0 (0.0%)				1 (100%)	0 (0.0%)	2 (1.9%)
Community network	1 (1.2%)	0 (0.0%)		1 (5.0%)	0 (0.0%)			2 (1.9%)
Family				1 (5.0%)	0 (0.0%)			1 (1.0%)
Total diagnosis Oncology = 49 Non-cancer = 47 No specification = 8	38 (45.8%)	37 (44.6%)	8 (9.6%)	10 (50%)	10 (50%)	1 (100%)	0 (0.0%)	
Total derivation Men = 56 (53.8%) Women = 48 (46.2% Age $\bar{X}$ = 74.3 years	83 (79.8%)			20 (19.2%)		1 (1%)		104 (100%)

* No pathology data (*n* = 7); ** No pathology data (*n* = 1).

**Table 2:** People in the initial and final support network.

	Initial Network		Final Network	Initial Network		Final Network
	Total (%)	Involve (%)	Involve (%)	$\bar{X}$ People	$\bar{X}$ Involved	$\bar{X}$ Involved
Family						
Internal network	306 (72.9%)	217 (76.1%)	295 (60.6%)	3.9	2.7	3.6
Non-family						
External network	74 (17.6%)	44 (15.4%)	117 (24.0%)	0.9	0.5	1.4
Community	40 (9.5%)	24 (8.4%)	75 (15.4%)	0.5	0.3	0.9
Total	420	285	487	5.4	3.6	6.1

**Table 3:** Type of needs in the initial network.

Type of Need	People without Need (%)	People in Need		
		Satisfied (%)	Not Satisfied (%)	Total Peolple in Need * (%)
Barthel				
To get ready	26 (32.1%)	55 (67.9%)	0 (0.0%)	55 (67.9%)
Bath (Wash)	22 (27.2%)	57 (70.4%)	2 (2.5%)	59 (72.8%)
To eat	32 (39.5%)	47 (58.0%)	2 (2.5%)	49 (60.5%)
Wandering	42 (51.9%)	34 (42.0%)	5 (6.2%)	39 (48.1%)
Deposition	49 (60.5%)	30 (37.0%)	2 (2.5%)	32 (39.5%)
Go to the toilet. Unable to access or use	54 (66.7%)	26 (32.1%)	1 (1.2%)	27 (33.3%)
Urination	50 (61.7%)	30 (37.0%)	1 (1.2%)	31 (38.3%)
Go up/down stairs	55 (67.9%)	19 (23.5%)	7 (8.6%)	26 (32.1%)
Transfer (bed/chair). Two people	40 (49.4%)	38 (46.9%)	3 (3.7%)	41 (50.6%)
Dress	25 (30.9%)	56 (69.1%)	0 (0.0%)	56 (69.1%)
Lawton and Brody				
Home care	8 (9.9%)	72 (88.9%)	1 (1.2%)	73 (90.1%)
Go shopping	10 (12.3%)	70 (86.4%)	1 (1.2%)	71 (87.7%)
Laundry	11 (13.6%)	69 (85.2%)	1 (1.2%)	70 (86.4%)
Money handling	30 (37.0%)	49 (60.5%)	2 (2.5%)	51 (63.0%)
Food preparation	35 (43.2%)	43 (53.1%)	3 (3.7%)	46 (56.8%)
Responsibility for your medication	24 (29.6%)	55 (67.9%)	2 (2.5%)	57 (70.4%)
Use of means of transport	45 (55.6%)	32 (39.5%)	4 (4.9%)	36 (44.4%)
Phone use	47 (58.0%)	30 (37.0%)	4 (4.9%)	34 (42.0%)
Others (emotional, accompaniment)				
Accompanied on medical visits	18 (22.2%)	60 (74.1%)	3 (3.7%)	63 (77.8%)
Accompanies the patient at home, hospital	4 (4.9%)	74 (91.4%)	3 (3.7%)	77 (95.1%)
Entertainment	33 (40.7%)	35 (43.2%)	13 (16.0%)	48 (59.3%)
Facilitates emotional release	28 (34.6%)	38 (46.9%)	15 (18.5%)	53 (65.4%)
Information management/communication with professionals, family	19 (23.5%)	62 (76.5%)	0 (0.0%)	62 (76.5%)
Manage visits, calls for consultation, emergencies	12 (14.8%)	69 (85.2%)	0 (0.0%)	69 (85.2%)
Wills	44 (54.3%)	4 (4.9%)	33 (40.7%)	37 (45.7%)

* Total people in need (Satisfied and Not satisfied).

**Table 4:** Type of needs in the final network.

Type of Need	People without Need (%)	People in Need		
		Satisfied (%)	Not Satisfied (%)	Total Peolple in Need * (%)
Barthel				
To get ready	14 (22.6%)	48 (77.4%)	0 (0.0%)	48 (77.4%)
Bath (Wash)	12 (19.4%)	49 (79.0%)	1 (1.6%)	50 (80.6%)
To eat	24 (38.7%)	37 (59.7%)	1 (1.6%)	38 (61.3%)
Wandering	34 (54.8%)	24 (38.7%)	4 (6.5%)	28 (45.2%)
Deposition	35 (56.5%)	26 (41.9%)	1 (1.6%)	27 (43.5%)
Go to the toilet. Unable to access or use	38 (61.3%)	24 (38.7%)	0 (0.0%)	24 (38.7%)
Urination	37 (59.7%)	24 (38.7%)	1 (1.6%)	25 (40.3%)
Go up/down stairs	47 (75.8%)	10 (16.1%)	5 (8.1%)	15 (24.2%)
Transfer (bed/chair). Two people	31 (50.0%)	28 (45.2%)	3 (4.8%)	31 (50.0%)
Dress	11 (17.7%)	51 (82.3%)	0 (0.0%)	51 (82.3%)
Lawton and Brody				
Home care	8 (12.9%)	53 (85.5%)	1 (1.6%)	54 (87.1%)
Go shopping	8 (12.9%)	54 (87.1%)	0 (0.0%)	54 (87.1%)
Laundry	7 (11.3%)	54 (87.1%)	1 (1.6%)	55 (88.7%)
Money management (Financial matters)	18 (29.0%)	42 (67.7%)	2 (3.2%)	44 (71.0%)
Food preparation	22 (35.5%)	39 (62.9%)	1 (1.6%)	40 (64.5%)
Responsibility for your medication	18 (29.0%)	43 (69.4%)	1 (1.6%)	44 (71.0%)
Use of means of transport	41 (66.1%)	18 (29.0%)	3 (4.8%)	21 (33.9%)
Phone use	31 (50.0%)	27 (43.5%)	4 (6.5%)	31 (50.0%)
Others (emotional, accompaniment)				
Accompanied on medical visits	14 (22.6%)	47 (75.8%)	1 (1.6%)	48 (77.4%)
Accompanies the patient at home, hospital	4 (6.5%)	58 (93.5%)	0 (0.0%)	58 (93.5%)
Entertainment	13 (21.0%)	45 (72.6%)	4 (6.5%)	49 (79.0%)
Facilitates emotional release	11 (17.7%)	47 (75.8%)	4 (6.5%)	51 (82.3%)
Information management/communication with professionals, family	10 (16.1%)	52 (83.9%)	0 (0.0%)	52 (83.9%)
Manage visits, calls for consultation, emergencies	7 (11.3%)	55 (88.7%)	0 (0.0%)	55 (88.7%)
Wills	28 (45.1%)	1 (1.6%)	33 (53.2%)	34 (54.8%)

* Total people in need (Satisfied and Not satisfied).

**Table 5:** Assessment of loneliness in the initial and final network.

Loneliness	Initial Network *n* = 57		Final Network *n* = 24	
	Average Punctuation	Number of People (%)	Average Punctuation	Number of People (%)
Short	8.1	18 (31.6%)	8	9 (37.5%)
Half	14.7	37 (64.9%)	14.1	14 (58.3%)
high	22	2 (3.5%)	21	1 (4.2%)
Total		57 (100%)		24 (100%)

**Table 6:** Assessment of quality of life. Initial and final network.

Mobility	I have no problem walking	I have some trouble walking.	I have to be in bed
Initial assessment	4 (15.4%)	17 (65.4%)	5 (19.2%)
Final assessment	4 (15.4%)	14 (53.8%)	8 (30.8%)
Personal care	I have no problems with Personal Care.	I have some trouble washing or dressing myself.	I am unable to wash or dress myself.
Initial assessment	2 (7.7%)	13 (50.0%)	11 (42.3%)
Final assessment	3 (11.5%)	11 (42.3%)	12 (46.2%)
Daily activities	I have no problems doing my daily activities.	I have some problems doing my daily activities.	Yes, I am unable to carry out my daily activities.
Initial assessment	2 (7.7%)	13 (50.0%)	11 (42.3%)
Final assessment	2 (7.7%)	10 (38.5%)	14 (53.8%)
Pain/Discomfort	I have no pain or discomfort.	I have moderate pain or discomfort.	I have a lot of pain or discomfort.
Initial assessment	16 (61.5%)	6 (23.1%)	4 (15.4%)
Final assessment	18 (69.2%)	7 (26.9%)	1 (3.8%)
Anxiety Depression	I am not anxious or depressed.	I am moderately anxious or depressed.	I am very anxious or depressed.
Initial assessment	12 (46.2%)	11 (42.3%)	3 (11.5%)
Final assessment	15 (57.7%)	10 (38.5%)	1 (3.8%)
Thermometer assessment (0 the worst imaginable state of health–100 the best imaginable state of health)			
Initial assessment	42.39		
Final assessment	45.29		
Score Quality of Life Scale 0 worst quality of life–1 best quality of life			
Initial assessment	0.34		
Final assessment	0.36		

## Data Availability

Not applicable.
